# Immature event-related alpha dynamics in children compared with the young adults during inhibition shown by day-night stroop task

**DOI:** 10.3389/fnhum.2023.1218559

**Published:** 2023-09-26

**Authors:** Bahar Güntekin, Simay Alptekin, Ebru Yıldırım, Tuba Aktürk, Hakan Uzunlar, Pervin Çalışoğlu, Figen Eroğlu Ada, Enver Atay, Ömer Ceran

**Affiliations:** ^1^Department of Biophysics, School of Medicine, Istanbul Medipol University, Istanbul, Türkiye; ^2^Research Institute for Health Sciences and Technologies (SABITA), Neuroscience Research Center, Clinical Electrophysiology, Neuroimaging and Neuromodulation Lab, Istanbul Medipol University, Istanbul, Türkiye; ^3^Department of Neuroscience, Graduate School of Health Sciences, Istanbul Medipol University, Istanbul, Türkiye; ^4^Program of Electroneurophysiology, Vocational School, Istanbul Medipol University, Istanbul, Türkiye; ^5^Department of Psychology, Humanities and Social Sciences, Istanbul Medipol University, Istanbul, Türkiye; ^6^Department of Pediatrics, School of Medicine, Istanbul Medipol University, Istanbul, Türkiye

**Keywords:** EEG, brain oscillations, event-related alpha oscillations, inhibition, maturation

## Abstract

**Introduction:**

Inhibitory control develops gradually from infancy to childhood and improves further during adolescence as the brain matures. Related previous studies showed the indispensable role of task-related alpha power during inhibition both in children and young adults. Nonetheless, none of the studies have been able to investigate the direct differences in brain responses between children and young adults when confronted with a stimulus that should be inhibited. Because, unlike event-related designs, task-related designs involve continuous tasks over a certain period, which precludes the possibility of making such a comparison. Accordingly, by employing event-related design, the present study first time in the literature, aimed to analyze the event-related alpha phase locking and event-related alpha synchronization/ desynchronization to differentiate the inhibitory processes in children compared to young adults.

**Methods:**

Twenty children between the ages of 6 to 7  years and 20 healthy young adult subjects between the ages of 18 to 30  years were included in the study. Day-night Stroop task was applied to all subjects during 18-channel EEG recordings. Event-related time-frequency analysis was performed with the complex Morlet Wavelet Transform for the alpha frequency band (8–13  Hz). Event related spectral perturbation (ERSP) in three different time windows (0–200  ms, 200–400  ms, 400–600  ms) and Event-related phase locking in the early time window (0–400  ms) was calculated.

**Results:**

The children had increased alpha power in early and late time windows but decreased alpha phase locking in the early time windows compared to young adults. There were also topological differences between groups; while young adults had increased alpha phase-locking in frontal and parietal electrode sites, children had increased occipital alpha power and phase locking.

**Discussion:**

The shift in event-related alpha power observed from posterior to anterior regions with age may suggest a progressive maturation of the frontal areas involved in inhibitory processes from childhood to adulthood. The results of the present study showed that children and young adults had different EEG oscillatory dynamics during inhibitory processes at alpha frequency range.

## 1. Introduction

Given the constantly evolving nature of our world, it is crucial to carefully choose the most suitable course of action to effectively achieve our objectives. In this context, inhibitory control is a pivotal component of executive functions, encompassing the capacity to successfully adapt to the environment to inhibit maladaptive behaviors, emotions, and thoughts (Diamond, 2013). Effective inhibitory processes are essential for healthy development in individuals from childhood through adulthood. Indeed, numerous studies have suggested that poor inhibitory control may be associated with a range of disorders, such as Attention-Deficit/Hyperactivity Disorder (ADHD) (Schachar et al., 2000; Woltering et al., 2013; Einziger et al., 2021), schizophrenia (Reilly et al., 2008; Galaverna et al., 2012), and Obsessive-Compulsive Disorder (OCD) (Penadés et al., 2007).

The maturation of the brain, particularly the frontal cortex, is closely linked with the development of inhibitory control (Wolfe and Bell, 2007a; Luna et al., 2015). As such, inhibitory control develops gradually from infancy to childhood and improves further during adolescence as the brain matures (Luna et al., 2004). Williams et al. (1999) conducted a study examining inhibitory processes across the lifespan (ages 6–81) using a task that required participants to stop prepotent responses. The authors reported that stopping speed becomes faster with increasing age throughout childhood, with limited evidence of slowing across adulthood. Their results were in good accordance with previous results where authors showed children with a mean age of 9.8 were 50 ms faster than the children with a mean age of 7.9 (Schachar and Logan, 1990) and 11 years old children were 40 ms faster at stopping than the 8-year-old children. Accordingly, the literature supports the notion that the inhibitory process improves from childhood to adolescence as a result of maturation (Schachar and Logan, 1990; Williams et al., 1999; Luna et al., 2004). However, it may gradually slow down across adulthood (see Williams et al., 1999). Keeping in mind these behavioral studies that show maturation-related differences in inhibitory processes, the current study will investigate how the maturation of inhibitory processes is reflected in event-related brain oscillations.

Although behavioral studies are commonly used to assess inhibitory control, studies have also been investigating electrophysiological markers of inhibition in infants and children. Inhibitory control could be investigated with EEG methodologies, among them; event-related potential (ERP) studies are highly used in the research of inhibitory control (see Ciesielski et al., 2004; Zinchenko et al., 2019; Einziger et al., 2021) ERPs have the advantage of identifying time during inhibitory control (Zinchenko et al., 2019; Einziger et al., 2021). However, it is also noted that ERPs could be hard to be studied in infants or early childhood; therefore, some researchers preferred to use task-related responses, where the precise time could not be followed, but this methodology could be used by comparing the baseline to task-related functions.

Studies utilizing ERP methods suggest that N2 and P3 responses observed in the frontal-central regions are indicative of inhibitory processes (Jonkman, 2006; Johnstone et al., 2007; Hämmerer et al., 2010; Huster et al., 2013; Knežević and Marinković, 2017; Richardson et al., 2018). More specifically, the N2 response is often linked with conflict monitoring, as its amplitude tends to increase in situations where two competing responses must be evaluated (e.g., requiring whether to respond to a stimulus or inhibit the response) (Donkers and van Boxtel, 2004; for review see Huster et al., 2013). The P3 response, on the other hand, is mainly linked to assessing the effectiveness of inhibitory performance (Bruin et al., 2001; for review see Huster et al., 2013). Based on the developmental process, studies have demonstrated that children exhibit higher levels of N2 response compared to young adults. The amplitude of N2 gradually decreases with increasing age (Todd et al., 2008; Buss et al., 2011; Richardson et al., 2018; Zinchenko et al., 2019), whereas the amplitude of P3 tends to increase with age (Jonkman, 2006; Richardson et al., 2018). Furthermore, studies have observed that N2 (Jonkman, 2006) and P3 responses (Hämmerer et al., 2010) in children are more prominent in the posterior regions of the brain. As the brain matures during development, these ERP components shift towards more frontal-central electrodes (Jonkman, 2006; Hämmerer et al., 2010). These posterior activations are interpreted as indicating additional neural activity due to the underdevelopment of the frontal cortex (Jonkman, 2006).

In addition to examining the N2 and P3 components, research has also investigated the N450 component in the context of inhibitory processes, particularly conflict monitoring. The N450 component, originating from neural activity in the anterior cingulate cortex (ACC) and frontal brain regions, is believed to be observed in cognitive tasks involving conflict processing and the suppression of irrelevant information (West et al., 2004, 2005; Larson et al., 2009). Besides the N450 component, studies utilizing cognitive tasks that require the resolution of conflicting information have observed activation in later time windows (West, 2003; Coderre et al., 2011; Naylor et al., 2012 for review see Heidlmayr et al., 2020). This particular activation has been assigned different names, such as the late positive component (LPC) (see Coderre et al., 2011), the late sustained potential (LSP) (see Naylor et al., 2012) and the conflict slow-potential (conflict SP) (see Larson et al., 2012). Although the precise role of this component is still ambiguous, it seems to contribute to conflict resolution process (West, 2003; Coderre et al., 2011; Naylor et al., 2012; for review see Heidlmayr et al., 2020). Although there are limited studies focusing on N450 and conflict SP in children, Larson et al.’s (2012) study revealed that children demonstrate a higher N450 response and conflict SP compared to young adults. On the other hand, researchers examined conflict adaptation skills by investigating the difference between “incongruent trial preceded by an incongruent trial” and “incongruent trial preceded by a congruent trial,” and obtained similar results in children and adults (Larson et al., 2012).

One alternative approach to examining inhibitory control is through time-frequency decomposition, although the literature in this area remains limited. Assessing inhibitory control in laboratory settings commonly involves using paradigms such as Go/No-Go and Stop-Signal. Additionally, Stroop-like paradigms are also used in inhibition literature (Gerstadt et al., 1994). Stroop-like paradigms encompass variations of the original Stroop task that exhibit similar interference effects but employ different stimuli (such as the classic Stroop Interference task, day-night Stroop task, and Yes-No Stroop task). Although the specific stimuli may vary, the fundamental principle remains unchanged, where conflict emerges between the attributes of the stimulus (Stroop, 1935). While slight differences exist between Go/No-Go and Stop-Signal, they both require inhibiting a prepotent response. The Go/No-Go and Stop-Signal tasks are closely associated with behavioral inhibition, primarily focused on motor responses, as compared to Stroop-like tasks. On the other hand, Stroop paradigms differ from the other paradigms mentioned above by specifically requiring interference control, which involves inhibiting automatic responses and selectively attending to less automatic ones (e.g., saying “day” when the picture represents night). Furthermore, Stroop paradigms appear more ecologically valid due to their resemblance to real-life situations where individuals must navigate and resolve conflicting information (Montgomery and Koeltzow, 2010). Since the day-night Stroop task is preferred in this research, the studies that have employed Stroop-like paradigm in children will be summarized in the following paragraph.

As reviewed by Bhavnani et al. (2021), eight studies analyzed Stroop-like paradigms (Yes-No task, day-night task) in children. In these studies, the alpha power spectrum was investigated in a task-related paradigm. The researchers analyzed the difference between baseline power and task-related power. They found increased task-related alpha power compared to baseline during Stroop-like tasks. In a series of studies [Wolfe and Bell (2004), Bell and Wolfe (2007), Wolfe and Bell (2007a,b), Wolfe and Bell (2014); for review see Bhavnani et al. (2021)] showed the importance of alpha oscillations during inhibitory processes in infants and children. Studies showed that alpha power increased from baseline to task in children both eight months age infants and 3-to-5 years old children during Stroop-like day-night tasks (Wolfe and Bell, 2007a,b, 2013). Cuevas et al. (2016) later confirmed these results and also showed increased alpha power during Stroop-like versus Non-Stroop-like day-night tasks.

These pioneering and essential studies showed the indispensable role of task-related alpha power during inhibition in children. Nonetheless, none of the studies have been able to investigate the direct differences in brain responses between children and young adults when confronted with a stimulus that should be inhibited. Because, unlike event-related designs, task-related designs involve continuous tasks over a certain period, which precludes the possibility of making such a comparison but investigates more like the status of the brain during the task. Accordingly, task-related approaches are limited in their ability to analyze event-related phase locking, event-related synchronization, and desynchronization. Having a precise zero point in EEG data, as in the event-related approach, is an important step in investigating neural correlates of inhibitory response to presented stimuli, directly. It gives us the opportunity to examine two important features of the stimulus-related time-locked brain signals; phase and power. Both features contribute valuable information about brain activity during event processing, but they capture different aspects of neural responses. Researchers often use these complementary approaches to gain a comprehensive understanding. By investigating phase-locking analysis, which measures the consistency of brain responses to stimuli and necessitates a precise zero point, we can infer the temporal precision of the brain’s response to the event. The phase-locked activity reflects the precise timing of neural responses relative to the event onset. The information related to phase and power represents distinct aspects of brain functioning, and as such, they may behave differently. For instance, Hanslmayr et al. (2005) demonstrated that visual discrimination performance was associated with a decrease in alpha power but an increase in phase locking. Consequently, the implementation of an event-related design to conduct event-related phase and power analysis can be essential in understanding how the brain processes sensory information or reacts to specific stimuli.

Therefore, along with the importance of task-related studies, event-related studies are quite needed in the field to achieve more robust results in this respect. To our knowledge, in all these studies, task-related alpha power was analyzed, but none of the studies investigated the event-related alpha power and phase locking in children with Stroop-like tasks by comparing with the young adults. Thus, the primary aim of this study is to evaluate the inhibitory process by comparing children and young adults using an event-related approach within the alpha frequency band. With this approach, it becomes possible to analyze phase-locking responses and synchronization/desynchronization in the alpha frequency band. In this regard, it is hypothesized that children and young adults would exhibit different brain topography in terms of event-related alpha dynamics. It is also expected to see differentiated alpha synchronization/desynchronization and phase-locking characteristics between children and young adults, reflecting the maturation-related developmental differences in inhibitory processes between the two groups.

## 2. Materials and methods

### 2.1. Participants

Twenty children between the ages of 6 to 7 years and 20 healthy young adult subjects between the ages of 18 to 30 years were included in the study. The demographic information of all subjects was given in Table 1. The participants were healthy subjects who had no neurological and/or psychiatric disorder and none of them use any neurological and/or psychiatric drugs, they had all normal or corrected vision with glasses or contact lenses. The Istanbul Medipol University Ethical Committee approved the present study (No: E34153). At the beginning of the study, the families (mother or father) of the children and all young subjects signed the informed consent form. At the end of the experiment, a toy car or doll was presented to the children as a gift. In addition, the attendance certificate was given to the children who completed the experiment successfully. The young volunteers did not receive any compensation for their participation.

**Table 1:** Demographic information and behavioral results of the children and the young adults.

	Children (*N* = 20) M ± SD	Young Adults (*N* = 20) M ± SD	*p*-value
Age (year)	6.65 ± 0.49	21.95 ± 2.58	**<0.001** ^**a**^
Education (year)	1.55 ± 0.51	15.45 ± 1.99	**<0.001** ^**a**^
Gender (F/M)	10 /10	10 / 10	**>0.05** ^**b**^
WM index scores	102.15 ± 11.48	–	
PS index scores	113.65 ± 12.10	–	
DNT error scores	4.85 ± 4.78	0.75 ± 1.33	**<0.001** ^**c**^

WM, working memory; PS, processing speed; DNT, day–night Stroop task; M, Mean; SD, Standard deviation; F, Female; M, Male.

a Mann–Whitney *U*.

b Chi-square.

c Kruskal–Wallis.

### 2.2. Neuropsychological tests

#### 2.2.1. Cognitive proficiency

Working Memory (WM) and Processing Speed (PS) subscales of the Wechsler Intelligence Scale for Children (WISC-IV; Wechsler, 2003), which was developed to assess the general intellectual functioning of children aged 6 to 16 years, were applied to children to measure children’s cognitive proficiency. These two subscales focus more on the adequacy and efficiency of cognitive processing and require good mental control (Weiss and Gabel, 2008). The normative mean score is 100 (SD = 15) for both subscales. The Turkish version of the WISC-IV (Oktem et al., 2016), a valid and reliable measurement tool, was used in the present study.

### 2.3. Procedure and task

Day-night Stroop task was applied to all subjects during EEG recording in the study. The day-night Stroop task is a cognitive paradigm that asses frontal inhibition, especially in children. The ability to inhibit a dominant response and to activate an alternative response is assessed with this paradigm (Gerstadt et al., 1994; Panesi and Morra, 2020). Two types of stimuli were applied in the day-night Stroop task: day and night stimuli. The sun picture represented the day stimuli, while the moon and stars picture represented the night stimuli. The participants were asked to say verbally “day” to the night stimuli when the moon and stars pictures were presented and “night” to the day stimuli when the sun pictures were presented on the screen. The researcher noted the verbal responses given to each stimulus during EEG recording. In the day-night Stroop task, there were 80 stimuli (day stimuli: 40, night stimuli: 40) in total. The “night” answer for the “night” stimuli and the “day” answer for the “day stimuli were considered as the wrong answers. Accordingly, the error scores of the participants were calculated based on their answers for the presented 80 stimuli in total. While the higher error scores indices failing response inhibition, lower error scores show successful inhibition.

The yellow sun picture on a white background was used for the day stimuli, and the yellow moon and stars picture on a white background was used for the night stimuli. The white background was used for two types of stimuli so that there was no difference between the luminance of the two types of stimuli as checked by the luminance meter (HD 2302.0 LightMeter). The luminance values were 114 cd/cm2 and 107 cd/cm2 for day and night stimuli, respectively. The stimuli were randomly presented by 1,000 ms duration at full size on a 21-inch computer monitor (refresh rate: 60 Hz). The inter-stimulus interval was 5 s.

### 2.4. EEG recording

Brain Vision Recorder (Brainproducts, Munich, Germany) software and BrainAmp MR Plus (Brainproducts, Munich, Germany) system machine were used for EEG recording. EEG recordings were performed with a sampling rate of 500 Hz, and the band limits were 0.01–250 Hz. EEG was recorded from 18 channels (F3, F4, C3, C4, P3, P4, O1, O2, T7, T8, TP7, TP8, P7, P8, Fz, Cz, Pz, Oz) using the gold (Au) disc electrodes. The electrodes were placed according to the International 10–20 system. In addition, two additional linked (A1 + A2) earlobe electrodes were used for referencing, and all electrodes were referenced to these two electrodes. The ground electrode was placed behind the right earlobe. EOG electrodes (HEOG and VEOG) were placed on the medial upper and lateral orbital rim of the right eye for the recording of the eye movements. All children standardly played with plastic construction toy blocks during the preparation phase (during the placing of the electrodes). If the children did not want to continue the experiments at any stage, the experiment terminated. The impedance values were below 10 kΩ (kiloohm) for all electrodes. EEG recordings were performed in a dimly-lighted and isolated room. The mother or a father sat behind the children during recordings, they were silent and not in the children’s visual area.

### 2.5. EEG analysis

#### 2.5.1. EEG data preprocessing

Brain Vision Analyzer (Brainproducts, Munich, Germany) software was used for EEG data preprocessing. Firstly, raw data digitally were filtered between 0.01 and 60 Hz using the IIR filters function (order: 8), and then, ICA (Independent Component Analysis) was applied to filtered data to remove eye blinking and eye movement-related components from the data. Later, continuous data were segmented into the 2 s epochs, which are 1,000 ms before stimulus onset and 1,000 ms after stimulus onset (−1,000 + 1,000 ms). The epochs which consisted of the correct responses only were included in the analysis. The remaining artifacts (muscle movement and electrode artifact etc.) were removed with the offline manual artifact rejection by carefully checking the data epoch by epoch. Finally, preprocessed EEG data was exported from Brain Vision Analyzer for time-frequency analyses.

#### 2.5.2. Analysis of the event-related spectral perturbation

Event-related spectral perturbation (ERSP) is one of the time-frequency measures and shows event-related changes in spectral power. In the present study, event-related power spectrum analysis was performed with the ERSP method. EEGLAB toolbox (Delorme and Makeig, 2004) was used to obtain total power by the ERSP analysis, and to do so preprocessed EEG data was imported to EEGLAB. In the ERSP analysis, complex Morlet Wavelet Transform was applied to data with 3 cycles width for the alpha frequency band (8–13 Hz). The frequency bin was chosen as 60. The values were transformed into the decibel (dB) scale. The reference interval for baseline correction was between −500 and − 300 ms time windows. In baseline correction, the mean amplitude in this reference interval was extracted from each data point after the stimulus. Later, the average power values were calculated in three different time intervals (0–200 ms), (200–400 ms), and (400–600 ms) at the alpha frequency band (8–13 Hz) for each participant, and these values were used for statistical analysis. The power analysis was performed at three different time points to see the event-related desynchronization in early and late time windows. The numerical values used for statistics were calculated using customized MATLAB scripts.

#### 2.5.3. Analysis of the event-related inter-trial coherence

Inter-trial coherence (ITC) is one of the time-frequency measures and is also called phase-locking analysis (Tallon-Baudry et al., 1996; Delorme and Makeig, 2004). ITC analysis shows the coherency of the phase angles between the event-related responses to each presented trial. In the present study, the event-related phase-locking analysis was performed by the EEGLAB toolbox (Delorme and Makeig, 2004), over the imported preprocessed EEG data. In the ITC analysis, complex Morlet Wavelet Transform was applied to data with 3 cycles width for the alpha frequency band (8–13 Hz). The frequency bin was chosen as 60. Later, the average phase-locking values were calculated in a particular time interval (0–400 ms) at the alpha frequency band (8–13 Hz) for each participant, and these values were used for statistical analysis. The numerical values used for statistics were calculated using customized MATLAB scripts. In the calculation of the numerical data used in statistics for both ITC and ERSP, the time windows were determined according to the grand averages of each group.

### 2.6. Statistical analysis

The statistical analyses were performed by repeated measures analysis of variance (ANOVA) by using Jamovi software (version 2.3.24). Separate statistical analyses were run for the ITC and ERSP. Accordingly, there were two different ANOVA designs for each. In the ANOVA design for ERSP, two different subject groups (children, and young adults) were included as a between-subject factor. Three different time intervals (0–200 ms), (200–400 ms), (400–600 ms), seven locations [frontal (F3-F4), central (C3-C4), parietal-1 (P3-P4), occipital (O1-O2), temporal (T7-T8), temporo-parietal (TP7-TP8), parietal-2 (P7-P8)], and two hemispheres (left, right) were included as within-subject factors. In the ANOVA design for ITC, two different subject groups (children, and young adults) were included as a between-subject factor. In addition, seven locations [frontal (F3-F4), central (C3-C4), parietal-1 (P3-P4), occipital (O1-O2), temporal (T7-T8), temporo-parietal (TP7-TP8), parietal-2 (P7-P8)] and two hemispheres (left, right) were included as within-subject factors. Greenhouse–Geisser corrected *p* values are reported. Bonferroni correction was used and reported in the pairwise comparisons for the *post-hoc* tests. Furthermore, group comparisons for age and education were performed with the Mann–Whitney-U test, and group comparisons for gender were performed with the Chi-square test. The significance level was determined as *p* < 0.05 for all comparisons. The Kruskal–Wallis test was used for the statistical analysis of the behavioral data. The Dwass-Steel-Critchlow-Fligner adjusted *p*-values were reported for the Kruskal–Wallis test. The descriptive statistics of WM and PS index scores were presented in Table 1 as mean and standard deviation.

## 3. Results

### 3.1. Neuropsychological and behavioral results

The average of the WM index scores of the children was 102.15 (*SD*: ± 11.48, min.: 85, max.: 132). The PS index scores were 113.65 points on average (*SD*: ± 12.10, min.: 88, max.: 135). It was shown that both WM and PS index scores of all children participating in the study were within or above the threshold of the normal intelligence range.

The number of incorrect responses during the day-night Stroop task were given in Table 1. As seen in Table 1, the groups differed significantly (*p* < 0.001) on the number of incorrect responses on the Stroop test with children making more mistakes (4.85 (SD: ± 4.78, min.: 0, max.: 20)) than adults (0.75 (SD: ± 1.33, min.: 0, max.: 5)).

### 3.2. Results of the event-related alpha responses

#### 3.2.1. Results of ERSP analysis

##### 3.2.1.1. Group comparisons

**Figure 1:**
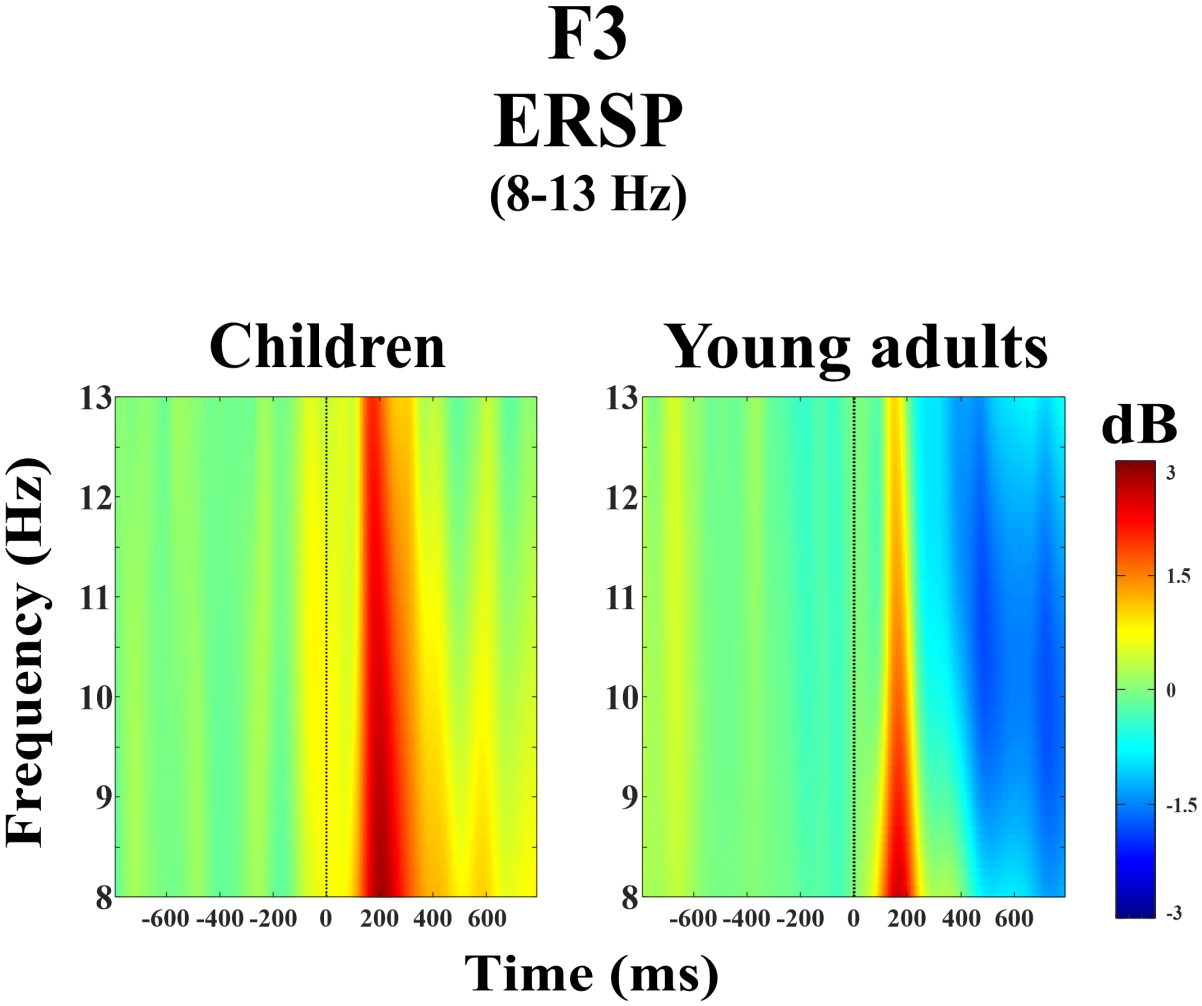
Grand average of ERSP in the alpha frequency band (8–13 Hz) for children and young adults over the F3 electrode.

The group main effect was statistically significant (*F*(1, 38) = 7.98, *p* = 0.008, η_p_^2^ = 0.174). Figure 1 represents the grand average of ERSP in the alpha frequency band (8–13 Hz) over the F3 electrode during the day-night Stroop task. The red color in the figure represented the higher ERSP values whereas the blue color the lower ERSP values. As seen in Figure 1, children had higher alpha power than young adults.

**Figure 2:**
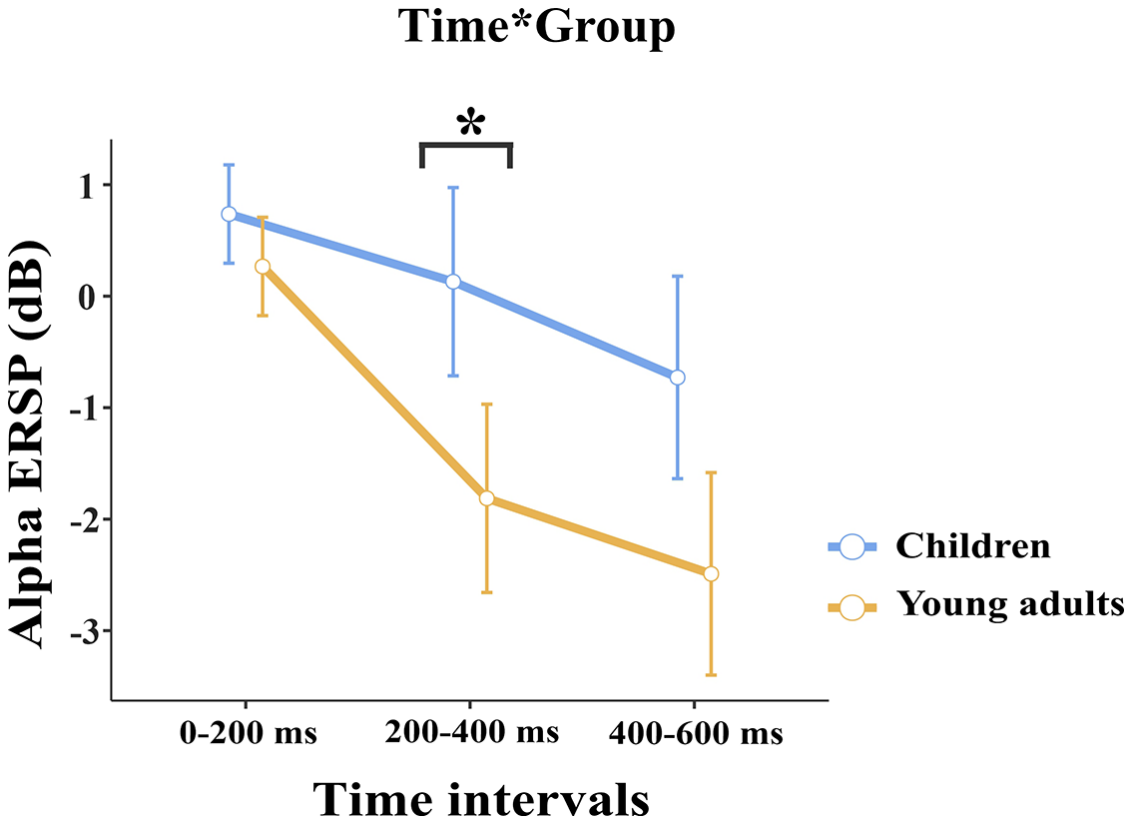
The statistical results of the alpha ERSP for the time interval × group interaction. The blue line represents the children, and the red line represents the young adults. In both 200–400 ms and 400–600 ms time intervals, children elicited higher alpha power than young adults. The error bars denote the 0.95 confidence interval. The asterisk sign indicates a statistically significant difference between groups for time intervals (*p < 0.05).

**Figure 3:**
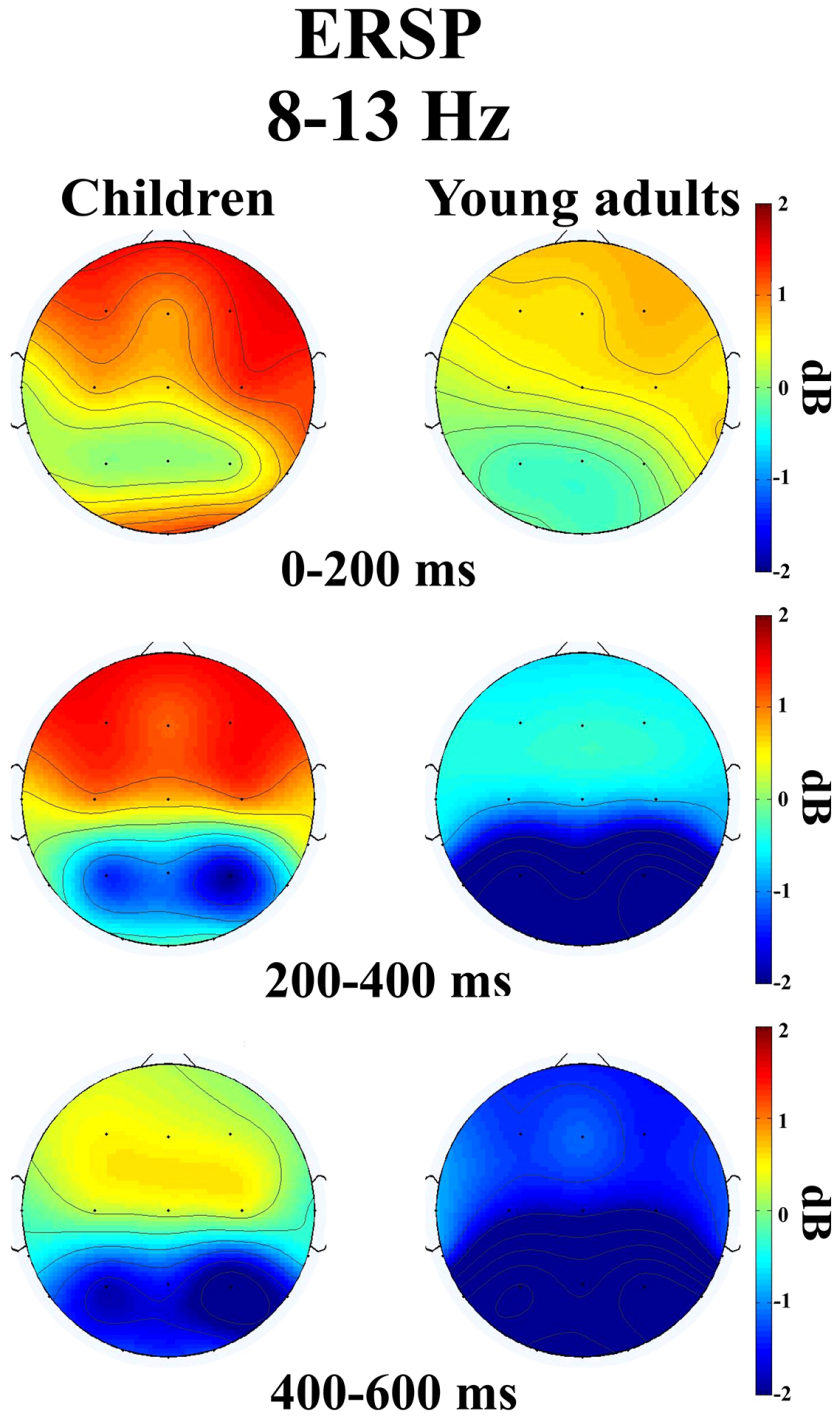
Topographical plots of the children and young adults in three different time intervals ((0–200 ms), (200–400 ms), and (400–600 ms)) for the alpha ERSP (8–13 Hz).

The groups differed significantly depending on time windows (*F*(1.32, 50.28) = 11.04, *p* < 0.001, η_p_^2^ = 0.225). *Post-hoc* comparisons showed that children had higher event-related alpha power than young adults within 200–400 ms time window (*t*(38.0) = 3.29, *p* = 0.032) (Figure 2). Figure 3 shows topographical plots of the children and young adults for the alpha ERSP within 0–200 ms, 200–400 ms, and 400–600 ms time windows. The red color in the figure represented the higher ERSP values whereas the blue color the lower ERSP values. As seen in Figure 3, children had higher event-related alpha power than young adults at all time windows. In the late time window, this difference was due to decreased alpha power seen in adults, but this decrease was not observed in children. The young adults had reduced alpha power in the late time window, which could be called alpha desynchronization (ERD).

**Figure 4:**
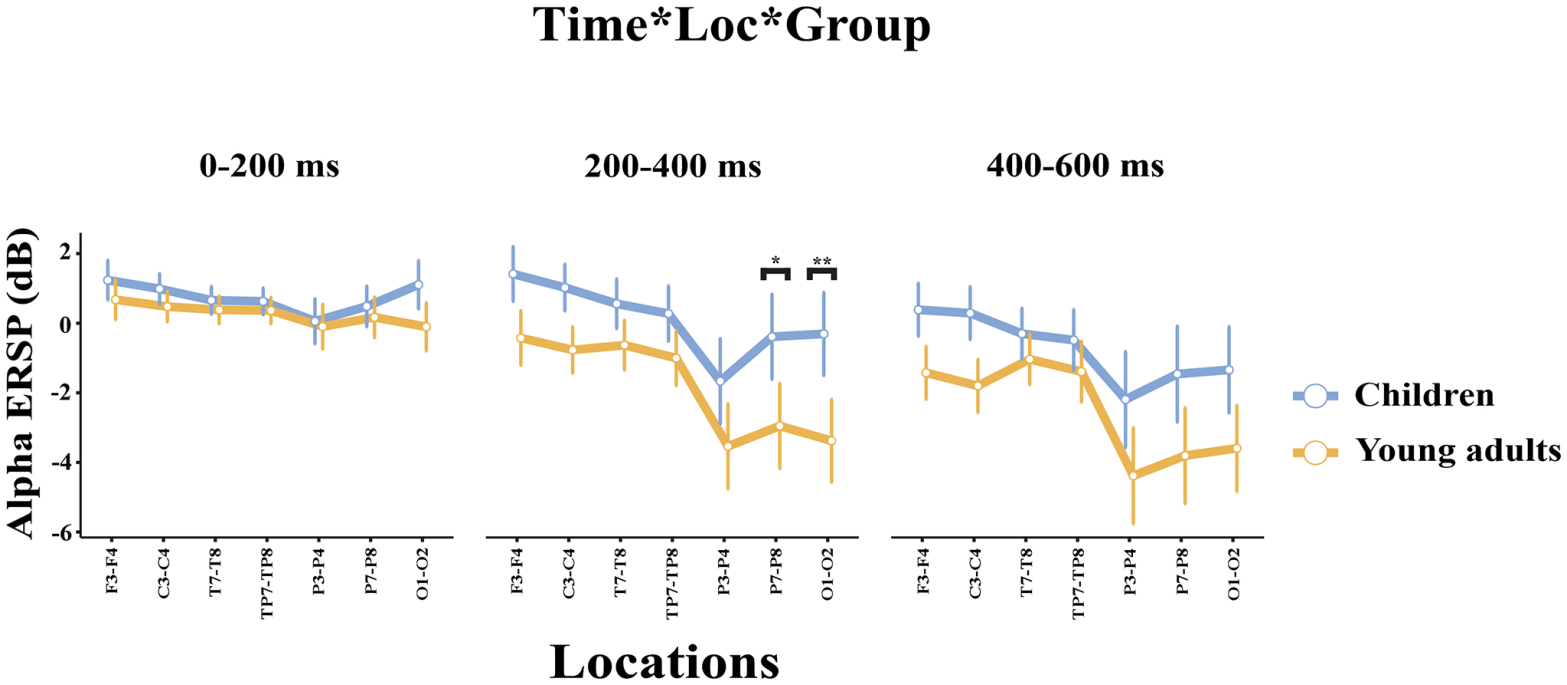
The statistical results of the alpha ERSP for the time interval × location × group interaction. The blue line represents the children, and the red line represents the young adults. The ERSP measures for the 0–200 ms time interval are represented in the left side of the picture, the ERSP measures within 200–400 ms time interval are represented in the middle part of the picture, and the ERSP measures within 400–600 ms time interval are represented in the right side of the picture. The error bars denote the 0.95 confidence interval. The asterisk signs indicate statistically significant differences between groups for time intervals (*p < 0.05; **p < 0.001).

The groups differed significantly depending on locations (*F*(2.47, 93.81) = 3.115, *p* = 0.039, η_p_^2^ = 0.076). Accordingly, children had higher event-related alpha power in the central locations than young adults (*t*(38.0) = 3.80, *p* = 0.046). The groups differed significantly depending on time windows and locations (*F*(3.46, 131.43) = 2.824, *p* = 0.034, η_p_^2^ = 0.069) (Figure 4). As seen in Figure 4, children elicited higher event-related alpha power than young adults in the late time window in the posterior locations.

##### 3.2.1.2. Within subject comparisons

The comparisons among time windows were statistically significant (*F*(1.32, 50.28) = 78.186, *p* < 0.001, η_p_^2^ = 0.673). *Post-hoc* comparisons showed that event-related alpha power within 0–200 ms time interval was higher than within 200–400 ms time interval (*t*(38.0) = 7.54, *p* < 0.001) and within 400–600 ms time interval (*t*(38.0) = 9.86, *p* < 0.001). Furthermore, event-related alpha power within 200–400 ms time interval was higher than within 400–600 ms time interval (*t*(38.0) = 7.69, *p* < 0.001).

There was a statistically significant difference among locations (*F*(2.47, 93.81) = 36.853, *p* < 0.001, η_p_^2^ = 0.492). *Post-hoc* analyses showed that alpha ERSP in the frontal locations was higher than in the parietal-1 (*t*(38.0) = 8.79, *p* < 0.001), parietal-2 (*t*(38.0) = 6.49, *p* < 0.001), and occipital locations (*t*(38.0) = 6.80, *p* < 0.001). The event-related alpha power in the central locations was higher than in the parietal-1 (*t*(38.0) = 8.52, *p* < 0.001), parietal-2 (*t*(38.0) = 5.60, *p* < 0.001), and occipital locations (*t*(38.0) = 5.54, *p* < 0.001). The alpha ERSP in the temporal locations was higher than in the temporo-parietal (*t*(38.0) = 3.63, *p* = 0.017), parietal-1 (*t*(38.0) = 7.99, *p* < 0.001), parietal-2 (*t*(38.0) = 5.48, *p* < 0.001), and occipital locations (*t*(38.0) = 5.30, *p* < 0.001). The alpha ERSP in the temporo-parietal locations was higher than in the parietal-1 (*t*(38.0) = 7.72, *p* < 0.001), parietal-2 (*t*(38.0) = 4.89, *p* < 0.001), and occipital locations (*t*(38.0) = 4.32, *p* = 0.002). In addition, the alpha ERSP in the parietal-2 and occipital locations was higher than in the parietal-1 location (*t*(38.0) = −4.93, *p* < 0.001; *t*(38.0) = −4.46, *p* = 0.001, respectively). The alpha ERSP in the frontal, central, temporal, and temporoparietal locations was higher than in the parietal and occipital locations. This was mostly due to the increased alpha ERD in parietal and occipital locations. The Time interval × Location interaction was statistically significant (*F*(3.46, 131.43) = 24.035, *p* < 0.001, η_p_^2^ = 0.387). *Post-hoc* comparisons showed that alpha ERSP within 0–200 ms time interval was higher than within 200–400 ms time interval in the temporo-parietal (*t*(38.0) = 4.67, *p* = 0.008), parietal-1 (*t*(38.0) = 9.25, *p* < 0.001), parietal-2 (*t*(38.0) = 7.15, *p* < 0.001), and occipital locations (*t*(38.0) = 7.90, *p* < 0.001). The alpha ERSP within 0–200 ms time interval was higher than within 400–600 ms time interval in the frontal (*t*(38.0) = 7.15, *p* < 0.001), central (*t*(38.0) = 6.66, *p* < 0.001), temporal (*t*(38.0) = 6.14, *p* < 0.001), temporo-parietal (*t*(38.0) = 6.18, *p* < 0.001), parietal-1 (*t*(38.0) = 9.48, *p* < 0.001), parietal-2 (*t*(38.0) = 8.44, *p* < 0.001), and occipital locations (*t*(38.0) = 8.66, *p* < 0.001). Furthermore, the alpha ERSP within 200–400 ms time interval was higher than within 400–600 ms time interval in the frontal (*t*(38.0) = 8.61, *p* < 0.001), central (*t*(38.0) = 5.96, *p* < 0.001), temporal (*t*(38.0) = 5.38, *p* < 0.001), temporo-parietal (*t*(38.0) = 4.39, *p* = 0.018), parietal-1 (*t*(38.0) = 4.75, *p = 0*.006), parietal-2 (*t*(38.0) = 6.33, *p* < 0.001), and occipital locations (*t*(38.0) = 4.10, *p* = 0.044).

The Time interval × Hemisphere interaction was statistically significant (*F*(1.82, 69.09) = 19.817, *p* < 0.001, η_p_^2^ = 0.343)). *Post-hoc* analyses showed that the alpha power of the right hemisphere within 0–200 ms time interval was higher than the left hemisphere *t*(38.0) = −3.67, *p = 0*.011). The Time interval × Location × Hemisphere interaction was statistically significant (*F*(5.47, 207.76) = 4.596, *p* < 0.001, η_p_^2^ = 0.108).

In summary, children had higher alpha power than young adults. Especially, the young adults had decreased alpha power on late time window and posterior locations than children due to ERD. However, this alpha ERD was not seen in children.

#### 3.2.2. Results of ITC analysis

##### 3.2.2.1. Group comparisons

**Figure 5:**
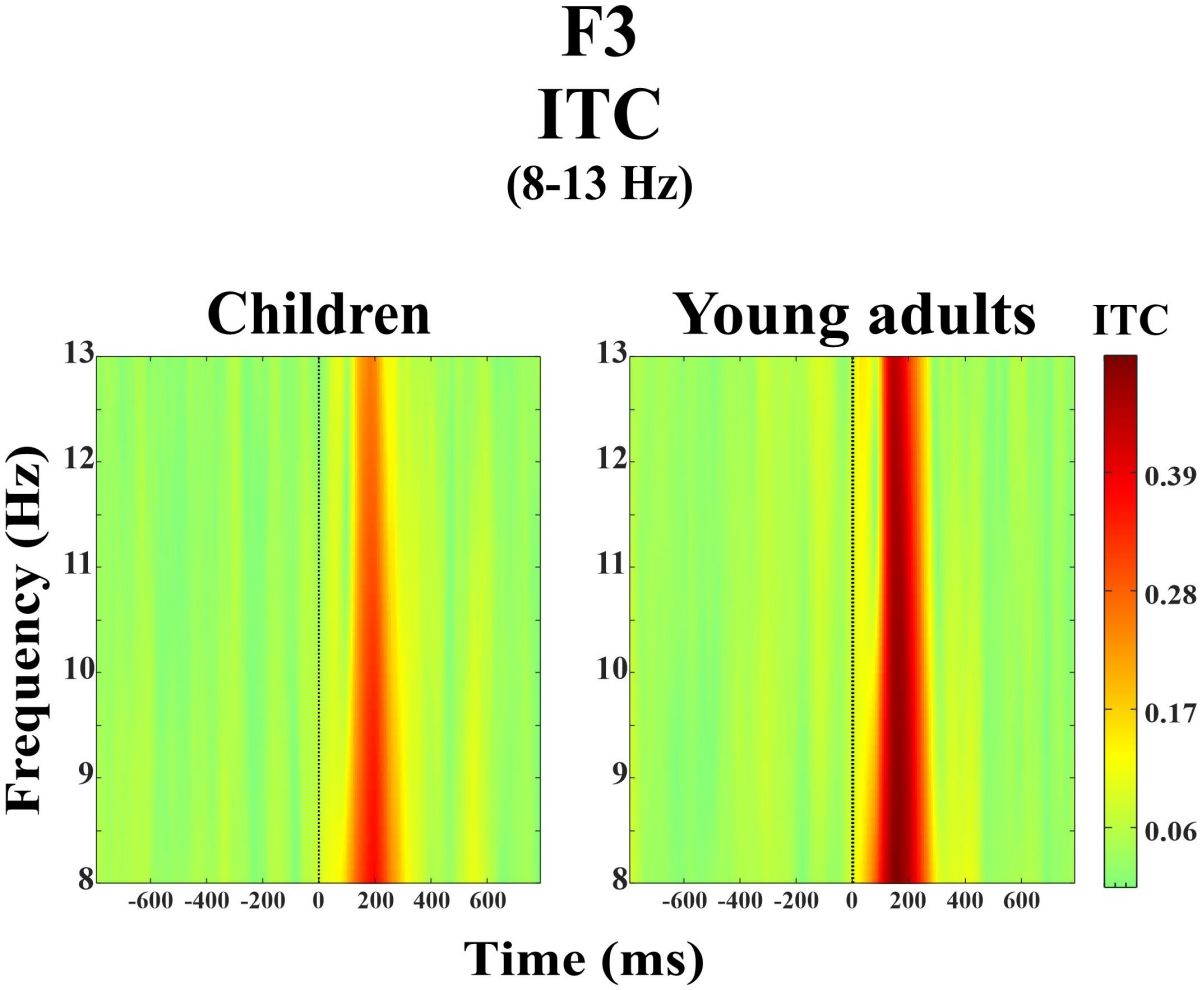
Grand average of ERSP in the alpha frequency band (8–13 Hz) for children and young adults over the F3 electrode.

The group main effect was statistically significant (*F*(1, 38) = 5.01, *p* = 0.031, η_p_^2^ = 0.116). Figure 5 represents the grand average of ITC in the alpha frequency band (8–13 Hz) over the F3 electrode during the day-night Stroop task. The red color in the figure represented the higher ITC values whereas the green color the lower ITC values. As seen in Figure 5, young adults had higher event-related alpha phase-locking than children.

**Figure 6:**
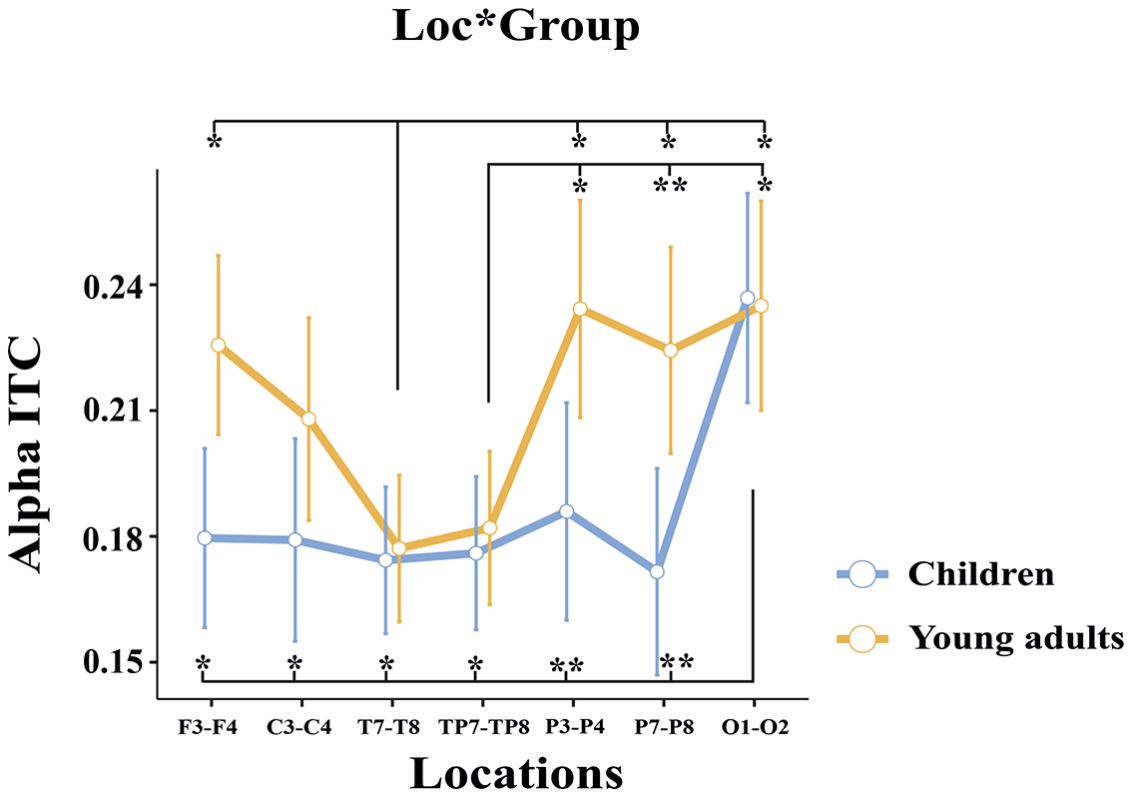
The statistical results of the alpha ITC for the location × group interaction. The blue ssline represents the children, and the red line represents the young adults. The error ssbars denote the 0.95 confidence interval. The asterisk signs indicate statistically significant differences between locations for groups (*p < 0.05; **p < 0.001).

The groups differed significantly depending on locations (*F*(3.35, 127.33) = 4.214, *p* = 0.005, η_p_^2^ = 0.100) (Figure 6). *Post-hoc* analyses showed that the alpha ITC of the children in the occipital location was higher than in the frontal (*t*(38.0) = −4.59, *p* = 0.004), central (*t*(38.0) = −3.78, *p* = 0.050), temporal (*t*(38.0) = −4.59, *p* = 0.004), temporo-parietal (*t*(38.0) = −4.93, *p* = 0.002), parietal-1 (*t*(38.0) = −5.63, *p* < 0.001), and parietal-2 locations (*t*(38.0) = −5.70, *p* < 0.001). Whereas the alpha ITC of the young adults in the frontal (*t*(38.0) = 4.63, *p* = 0.004), parietal-1 (*t*(38.0) = −4.43, *p* = 0.007), parietal-2 (*t*(38.0) = −4.32, *p* = 0.010), and occipital locations (*t*(38.0) = −4.25, *p* = 0.012) were higher than in the temporal location. In addition, the alpha ITC of the young adults in the parietal-1 (*t*(38.0) = −4.78, *p* = 0.002), parietal-2 (*t*(38.0) = −5.86, *p* < 0.001), and occipital locations (*t*(38.0) = −4.29, *p* = 0.011) were higher than in the temporo-parietal location.

##### 3.2.2.2. Within subject comparisons

The comparisons among locations were statistically significant (*F*(3.35, 127.33) = 12.490, *p* < 0.001, η_p_^2^ = 0.247). *Post-hoc* analyses showed that the alpha ITC in the occipital locations was higher than in the frontal (*t*(38.0) = −3.78, *p* = 0.011), central (*t*(38.0) = −3.92, *p* = 0.008), temporal (*t*(38.0) = −6.25, *p* < 0.001), temporo-parietal (*t*(38.0) = −6.52, *p* < 0.001), parietal-1 (*t*(38.0) = −4.04, *p* = 0.005), and parietal-2 (*t*(38.0) = −4.68, *p* < 0.001) locations. The alpha ITC in the frontal (*t*(38.0) = 3.63, *p* = 018) and parietal-1 locations (*t*(38.0) = −3.77, *p* = 0.012) was higher than in the temporal location. In addition, alpha ITC in the parietal-1 (*t*(38.0) = −4.03, *p* = 0.005) and and parietal-2 locations (*t*(38.0) = −3.71, *p* = 0.014) was higher than in the temporo-parietal location. There was statistically a significant difference between hemispheres (*F*(1, 38) = 13.846, *p* < 0.001, η_p_^2^ = 0.267). Accordingly, the event-related alpha phase-locking of the right hemisphere was higher than the left hemisphere (*t*(38.0) = −3.72, *p* < 0.001). The Location X Hemisphere interaction was statistically significant (*F*(4.04, 153.64) = 3.224, *p* = 0.014, η_p_^2^ = 0.078).

In summary, young adults had higher event-related alpha phase-locking than children. Furthermore, the groups differed topologically. The young adults had higher alpha phase-locking in frontal and parietal locations, whereas children had higher alpha phase-locking in the occipital location.

**Figure 7:**
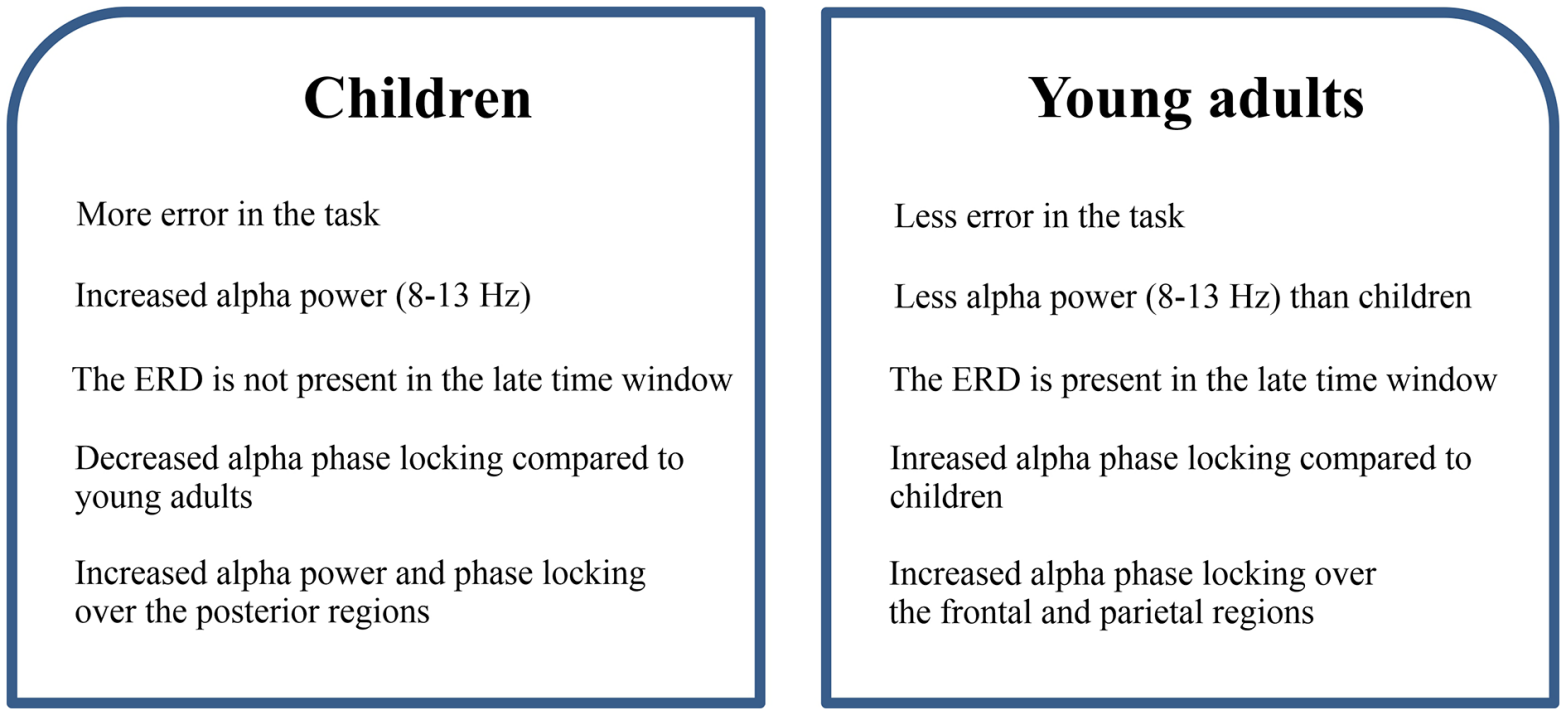
The summary of the results regarding child and adult differences.

The main results of the study are summarized in Figure 7.

## 4. Discussion

The main aim of the current study was to investigate the differences in inhibitory processes between 6-to-7-year-old children and young adults represented by the event-related inter-trial coherence (phase locking analysis) and event-related synchronization/desynchronization (power analysis) specifically in the alpha frequency band. To achieve this goal, we recorded EEG signals from all participants during the day-night Stroop task. As far as we know, this is the first study to explore these differences using an event-related approach.

The current study has been able to reveal some main differences between children and young adults in inhibitory processes, as depicted in Figure 7. Accordingly, behavioral results of the study revealed that young adults exhibited fewer errors compared to children. This finding is consistent with earlier studies in which inhibitory processes across different ages were investigated (Schachar and Logan, 1990; Williams et al., 1999). The EEG results from our study corroborate the differences observed in behavioral findings between children and young adults, which will be elaborated and discussed in the following paragraphs.

In the early and late time windows, our EEG results showed that children exhibited greater event-related alpha power than young adults. In the late time window, this difference was due to decreased alpha power seen in adults, which was not observed in children. In line with our findings, the study conducted by Sato et al. (2018) also found increased alpha power in children during working memory paradigm. Specifically, they observed increased alpha power in the left inferior parietal lobe after stimulus onset, which decreased after the retention interval. The authors suggested that this increase in alpha power could be due to the suppression of the dorsal stream, consistent with the inhibition hypothesis, this may reflect top-down attentional and/or inhibitory control (Klimesch et al., 2007; Jensen and Mazaheri, 2010). According to the inhibition hypothesis, the increase in alpha is considered to be the suppression of processing that is not necessary to perform the given task, while the alpha suppression is construed as cortical engagement (Pfurtscheller and Da Silva, 1999; Klimesch et al., 2007; Jensen and Mazaheri, 2010). In our study, the higher alpha activation observed in the early time windows, followed by the absence of event-related desynchronization (ERD) in children, could also be explained in terms of the inhibition hypothesis. Thus, the higher alpha response observed in children compared to young adults may be attributed to their increased need for resources and/or effort in order to suppress internal and external distractions. Similarly, as a continuation of this, the alpha decrease observed in young adults as a sign of cortical engagement has not been observed in children, which may be due to insufficient maturity of the top-down attentional processes in children.

On the other hand, in our previous study, increased alpha power and alpha phase locking values were shown in children during the visual and auditory item memory task (Güntekin et al., 2020). The authors discussed increased posterior alpha responses in children, as a compensation mechanism for immature top-down processes that coincide with overactive sensory processing. Additionally, several studies showed that alpha power can increase over parietal-occipital regions during tasks that require sensory processing, visual attention, or emotional processing (Güntekin and Basar, 2007; Başar, 2012; Başar and Güntekin, 2012). At this point, it is crucial to recognize that there are multiple alpha networks that may be related to different cognitive functions, including sensory processing, inhibition, memory processes, attention, and emotion. Since both above-mentioned studies (Sato et al., 2018; Güntekin et al., 2020) require sensory processing along with increased visual attention, even though the paradigms used in the two studies are different, it is possible that the increase in alpha power in parietal-occipital areas in children could be due to overly active sensory processing along with the visual attention during task performance. In accordance with these studies, in our study, the day-night Stroop task was presented to children through a computer as visual stimulation, which might have resulted in overactive perceptual processing and increased visual attention compared to healthy adults, potentially leading to increased parietal-occipital alpha responses in children. It remains unclear whether the observed increase in alpha power in children is solely attributable to overactive sensory processing, increased visual attention, or due to the suppression of the dorsal stream. A comparative investigation of different paradigms in children could shed light on the underlying mechanisms of these observed alpha dynamics, thereby advancing our understanding of the neural differentiations underlying sensory processes, visual attention, and cognitive control during development.

While the alpha power increased in children, as a complimentary of this finding, their alpha phase locking responses were lower than those observed in young adults. It seems that brain responses to the same type of stimulus are not consistent in children as reflected by lower alpha phase locking values. When we consider these main findings together, it can be said that the brain, children were not able to give a consistent response to the same type of stimuli that should be inhibited, as in young people, instead increased the strength of the EEG response as compensation. These findings also support a lack of maturation in inhibitory processes in children as required by the applied task.

The current study also indicates a topological shift from posterior to anterior regions during inhibitory processing from childhood to adulthood. Specifically, our results demonstrate greater alpha activation in the occipital and parietal regions in children compared to young adults. Our previous study reported not only increased activity over parietal and occipital areas for alpha responses but also similar results for theta responses during an item memory task (Güntekin et al., 2020). It was shown that young adults exhibited higher frontal theta power and phase-locking over frontal-central locations which were not observed in children who had reduced frontal responses (Güntekin et al., 2020). This posterior-to-anterior change appears to be due to the underdevelopment of the frontal areas or the heightened activity of the perceptual system in children, as discussed in the previous paragraph. As Jonkman (2006) suggested, the activation observed in posterior regions in children might indicate the need for additional neural activity due to the lack of maturation in the frontal cortex. On the other hand, Ofen and Shing (2013) have suggested that during childhood, episodic encoding may rely more on perceptual systems, while during adulthood, top-down frontal control becomes more prominent. It is possible that the perceptual system is more active in children than in adults, which could be reflected in increased alpha responses in children during inhibitory processes. Even though the exact mechanism remains a mystery, it appears that children have inverse frontal-occipital dynamics compared to young adults.

Although some studies have reported hemispheric differences shifting from left to right with age (see Bunge et al., 2002; Johnstone et al., 2005), our current study did not find any significant hemispheric differences between children and young adults. However, regardless of group, we did observe right hemisphere dominance in the early time windows. As we progress into the subsequent time windows, the right hemisphere dominance appears to decrease. This change may be related to approaching the time of verbal response. Existing research indicates that during the inhibitory process, adults generally display greater activation in the right hemisphere compared to the left hemisphere (Garavan et al., 1999; Aron et al., 2004), suggesting a potential link between the right hemisphere and competence in inhibition. However, due to the nature of our experimental paradigm, involving semantic processing, a competitive interaction may arise between cognitive processes that require verbal responses and inhibitory processes during the late time windows. Consequently, the dominant activation observed in the right hemisphere during early time windows might result from the conflict arising from the attributes of the stimulus and the requirement to inhibit automatic responses, along with selective attention to less automatic ones. Conversely, once this conflict is resolved, it appears that the involvement of the left hemisphere increases, as it takes a more active role in generating verbal responses, such as uttering “day” or “night.” Our findings also revealed that irrespective of the participant group, the response in the right hemisphere exhibited greater phase-locking compared to the left hemisphere. This result is consistent with the known association of the right hemisphere with inhibitory processes and hence, not unexpected.

All in all, our findings of increased event-related alpha power in children during the inhibitory task are consistent with task-related studies (Wolfe and Bell, 2004, 2007a, b, 2014; Bell and Wolfe, 2007). The current study also provides insights into alpha event-related synchronization/desynchronization and phase-locking differences between 6 and 7 years-old children and young adults using an event-related approach during the inhibitory processing.

The present study also had some limitations. The day-night Stroop task involves multiple cognitive processes, including memory, visual attention, decision-making, response inhibition, and motor processes related to the verbal expression of the answer. These processes are combined within a swift time window, making it difficult to attribute the observed oscillatory dynamics to the inhibition process solely. While the main function is inhibition, attention, decision-making, and motor response may also play a role in the observed alpha synchronization and desynchronization. However, it is important to acknowledge that avoiding this trade-off is also challenging for many cognitive neuroscience studies, as almost all tasks used in this field require some level of attentional and perceptual mechanisms. Nevertheless, future research could compare paradigms that may separate and manipulate specific cognitive functions to understand the underlying processes better. Other points that should be mentioned are the relatively small sample size of the study and the small number of electrodes utilized, which were enough electrodes for the employed analysis methods here, but, insufficient for source localization analysis like LORETA and Beamformer analysis. Another limitation of the current study was the analysis that was limited to the alpha frequency band. Although we tried to test a specific hypothesis related to the alpha band, it would be highly informative to examine the results of other frequency bands to ascertain the potential involvement of other frequencies in this inhibition phenomenon. Future studies should take these points into consideration to explore how other frequencies and neural localizations are implicated in this inhibition phenomenon. This could be achieved by increasing the participant number to enhance statistical power and facilitate robust conclusions.

## 5. Conclusion

The current study was conducted to elucidate the neural processes underlying inhibitory control in children and young adults. This study reveals that, concerning the inhibitory process, alpha dynamics in children differ from those of young adults. These differences are represented by increased alpha power in early time windows, decreased event-related desynchronization (ERD) in later time window, and phase-locking characteristics of alpha oscillations in children. In addition to these, the observed shift in event-related alpha power from posterior to anterior regions with age appears to align with a progressive maturation of the frontal areas from childhood to adulthood (Luna et al., 2004; Wolfe and Bell, 2007a). However, the reason why additional posterior brain regions are involved in inhibitory processes is still not well understood. This may stem from the perceptual system being more active in children (Sato et al., 2018; Güntekin et al., 2020), as well as the need for additional neural activity due to the incomplete maturation of the frontal cortex (Jonkman, 2006). Even though further studies are required to fully comprehend the underlying reasons, our study clearly demonstrates that children exhibit an inhibitory mechanism that has not yet fully developed, as evidenced by both behavioral and electrophysiological outcomes when compared to young adults.

## Data availability statement

The raw data supporting the conclusions of this article will be made available by the authors, without undue reservation.

## Ethics statement

The studies involving humans were approved by Istanbul Medipol University Ethics Committee. The studies were conducted in accordance with the local legislation and institutional requirements. Written informed consent for participation in this study was provided by the participants’ legal guardians/next of kin.

## Author contributions

ÖC, BG, TA, EY, FA, and EA initiated and planned the study and designed the protocol. BG, ÖC, SA, TA, EY, FA, and EA wrote, reviewed, and edited the paper. TA, EY, HU, and PÇ collected and analyzed the data. ÖC and BG supervised and controlled the study. All authors contributed to the manuscript revision, read and approved the submitted version.

## Conflict of interest

The authors declare that the research was conducted in the absence of any commercial or financial relationships that could be construed as a potential conflict of interest.

## Publisher’s note

All claims expressed in this article are solely those of the authors and do not necessarily represent those of their affiliated organizations, or those of the publisher, the editors and the reviewers. Any product that may be evaluated in this article, or claim that may be made by its manufacturer, is not guaranteed or endorsed by the publisher.
